# Dose-efficient multimodal microscopy of human tissue at a hard X-ray nanoprobe beamline

**DOI:** 10.1107/S1600577522001874

**Published:** 2022-03-16

**Authors:** Simone Sala, Yuhe Zhang, Nathaly De La Rosa, Till Dreier, Maik Kahnt, Max Langer, Lars B. Dahlin, Martin Bech, Pablo Villanueva-Perez, Sebastian Kalbfleisch

**Affiliations:** a MAX IV Laboratory, Lund University, 22100 Lund, Sweden; b Division of Synchrotron Radiation Research and NanoLund, Department of Physics, Lund University, 22100 Lund, Sweden; c Department of Medical Radiation Physics, Clinical Sciences Lund, Lund University, 22185 Lund, Sweden; d Excillum AB, 16440 Kista, Sweden; e Univ Lyon, INSA-Lyon, Université Claude Bernard Lyon 1, UJM-Saint Etienne, CNRS, Inserm, CREATIS UMR 5220, U1206, 69621 Villeurbanne, France; f Department of Translational Medicine – Hand Surgery, Lund University, Malmö, Sweden; g Department of Hand Surgery, Skåne University Hospital, Malmö, Sweden

**Keywords:** X-ray microscopy, in-line holography, X-ray fluorescence emission spectroscopy

## Abstract

X-ray fluorescence microscopy performed at nanofocusing synchrotron beamlines produces quantitative elemental distribution maps at unprecedented resolution (down to a few tens of nanometres), at the expense of relatively long measuring times and high absorbed doses. In this work, a method was implemented in which fast low-dose in-line holography was used to produce quantitative electron density maps at the mesoscale prior to nanoscale X-ray fluorescence acquisition. These maps ensure more efficient fluorescence scans and the reduction of the total absorbed dose, often relevant for radiation-sensitive (*e.g.* biological) samples. This multimodal microscopy approach was demonstrated on human sural nerve tissue. The two imaging modes provide complementary information at a comparable resolution, ultimately limited by the focal spot size. The experimental setup presented allows the user to swap between them in a flexible and reproducible fashion, as well as to easily adapt the scanning parameters during an experiment to fine-tune resolution and field of view.

## Abstract 2

This work presents the implementation of combined high-resolution X-ray in-line holography and X-ray fluorescence microscopy within the same experimental setup at a hard X-ray nanofocusing beamline. In-line holography provides morphological information by recovering electron density maps, even on weakly scattering or low-contrast samples; X-ray fluorescence provides complementary chemical information by producing element-specific mass density maps.

## 1. Introduction

The application of synchrotron-based X-ray microscopy techniques for the investigation of samples relevant for biomedical research has been increasing steadily in recent years. A major drive for this has been the wider availability of high-brilliance X-ray sources brought by the establishment of third- (Kunz, 2001 ▸; Bilderback *et al.*, 2005 ▸) and later fourth-generation synchrotron radiation facilities (Eriksson *et al.*, 2014 ▸), which deliver unprecedented high flux of coherent photons. As a result, several microscopy techniques have been developed and optimized exploiting different imaging modalities.

Synchrotron-based X-ray fluorescence (XRF) emission spectroscopy is a well established non-destructive microscopy technique (Paunesku *et al.*, 2006 ▸; Majumdar *et al.*, 2012 ▸; Cotte *et al.*, 2018 ▸) which generates quantitative elemental distribution maps with a resolution limited by the X-ray beam size. It is applied for the chemical investigation of samples from a wide range of research fields. In biology and medicine it proved useful within studies on both non-human (Dučić *et al.*, 2011 ▸; Victor *et al.*, 2018 ▸; Deng *et al.*, 2018 ▸; Silva Barreto *et al.*, 2020 ▸) and human (Fus *et al.*, 2019 ▸; Pascolo *et al.*, 2019 ▸; Carmona *et al.*, 2019 ▸; Conesa *et al.*, 2020 ▸) cells and tissues. The main benefit of performing XRF experiments at synchrotron radiation facilities is the high spatial resolution enabled by increasingly tight X-ray focal spot sizes, along with the high throughput enabled by the high photon flux. Owing to the latter, synchrotron-based XRF also features high sensitivity, being able to detect trace elements (down to p.p.m. concentration). On the other hand, tighter focus and higher flux imply higher absorbed dose, which can induce changes in the sample morphology and elemental distribution through radiation damage, thus negatively affecting the results’ reliability. While in practice the dose delivered during high-resolution XRF measurements is often experimentally constrained (*cf* *Discussion* [Sec sec4]), the dose delivered before that – for coarse sample navigation, for instance – can be more flexibly tuned and its minimization is often sought to prevent radiation damage, especially when dealing with radiation-sensitive biomedical samples.

Another broad group of X-ray microscopy techniques is that of coherent imaging (Mokso *et al.*, 2007 ▸; Chapman & Nugent, 2010 ▸; Mayo *et al.*, 2012 ▸; Langer *et al.*, 2012 ▸; Pfeiffer, 2018 ▸). These techniques can generate quantitative phase images, easily translated into quantitative electron density maps and providing direct insight into sample structure and morphology. As for XRF, these techniques have undergone significant spread and development with the rise of high-brilliance synchrotron radiation sources, although in this case it is mainly due to the increasingly high coherence of the available photon beams (Lewis, 2004 ▸; Momose, 2005 ▸). Elements with low atomic number (*Z*) feature weak X-ray absorption, implying that for organic materials – such as polymers and most biogenic samples – attenuation contrast due to small variations in electron density is poor. Such variations are better characterized by exploiting phase shift instead. Therefore, applying coherent X-ray imaging techniques to, for example, cells and tissues allows us to achieve better contrast and higher resolution with respect to absorption-contrast techniques. This can also be exploited to reduce absorbed dose and explains the widespread use of coherent imaging in research involving biological samples. One such coherent X-ray imaging technique is propagation-based in-line holography (Cloetens *et al.*, 1999 ▸). Similarly to XRF, in-line holography provides spatial resolutions limited by the focal spot size and is often found to be well suited to bio­medical samples (Bartels *et al.*, 2015 ▸; Töpperwien *et al.*, 2018 ▸; Dahlin *et al.*, 2020 ▸).

XRF measurements are sometimes combined with phase-contrast imaging within the same study, as these techniques return complementary information which can enhance overall results. Within such multimodal studies, phase contrast images can be obtained, for example, through holography (Veselý *et al.*, 2021 ▸), ptychography (Deng *et al.*, 2018 ▸; Stachnik *et al.*, 2020 ▸) or scanning transmission X-ray microscopy (STXM) (Li *et al.*, 2019 ▸; Pattammattel *et al.*, 2020 ▸). This latter approach is often carried out at the same beamline used for the XRF measurements as both techniques rely on a tightly focused X-ray beam. Furthermore, some STXM-based acquisition schemes have been tailored to reduce the total dose delivered to the sample through compressive sensing approaches (Kourousias *et al.*, 2020 ▸, 2021 ▸). In the case of holography, fewer beamlines combine it with XRF within the same setup and the correlation between the two imaging modes is carried out after the experiment (Cagno *et al.*, 2017 ▸; Lehmann *et al.*, 2019 ▸; Altenbach *et al.*, 2020 ▸; Gramaccioni *et al.*, 2020 ▸).

Here, we demonstrate the implementation of a multimodal approach which combines within the same experimental setup nanoscale XRF (nXRF) and in-line holography. Phase contrast images obtained with holography are used for selecting regions of interest (ROIs) for nXRF measurements, thus optimizing the measuring strategy while the experiment is ongoing. Our demonstration is carried out at the hard X-ray nanoprobe beamline NanoMAX (Johansson *et al.*, 2021 ▸) at MAX IV (Tavares *et al.*, 2014 ▸), which in 2016 began operation as the first of a new generation of synchrotron radiation sources based on diffraction-limited storage rings. Producing X-rays with a focal spot size down to below 50 nm (Björling *et al.*, 2020 ▸), NanoMAX is optimal for hard X-ray nXRF experiments and the high-coherent photon flux it delivers is well suited for coherent X-ray imaging. The combination of these two factors puts NanoMAX in an ideal position for driving the development of experiments which require such imaging modes. The two techniques were used to produce complementary 2D maps of the same ROIs within an osmium-stained section of a human peripheral sural nerve. The sample was chosen to be representative of how biomedical samples are prepared and behave under X-ray irradiation as the main goal of this study is to demonstrate its applicability to the wider research field. For every potential ROI, first, a low-dose scan was carried out by collecting free-space-propagated holograms of the sample positioned at different known defocusing distances in the divergent X-ray beam. This produced a near real-time 2D overview. Then, a more accurate ROI was selected therein and a longer XRF scan was carried out with the sample at a near-focus position. This produced a nanometric resolution map of the projected area mass density (*i.e.* mass per unit area) of the excited elements. Overall, our approach produces both projected electron density and elemental area mass density maps with comparable resolution. Furthermore, our approach allows us to minimize absorbed dose – and hence radiation damage – owing to the optimization of the field of view, based on the overview image.

## 2. Materials and methods

### 2.1. Sample

A whole peripheral sural nerve biopsy was performed on a healthy (male, age 60–65) living donor from the lower extremity (Sundkvist *et al.*, 2000 ▸; Mohseni *et al.*, 2017 ▸). The biopsy is a part of a larger study and sample preparation was carried out following the protocol described in detail by Sundkvist *et al.* (2000 ▸). In brief, the sural nerve biopsy was fixed in 0.1 *M* cacodylate-buffered (pH 7.3) 2.5% glutaraldehyde. The biopsy was rinsed and post-fixed in 1% osmium tetroxide [4% sucrose, 1.5% K_3_Fe(CN)_6_ in cacodylate buffer]. The specimen was then dehydrated via graded series of ethanol solutions (50–100%) and finally infiltrated with propylene oxide prior to embedding in epoxy resin (Epon 812). After these processes, routinely carried out for electron microscopy analysis, 2 µm-thin sections were produced using an ultramicrotome (Leica EM UC7, Leica Microsystems, Germany) equipped with a histo diamond knife (DiATOME, USA). The sections were deposited onto 1 mm × 1 mm, 200 nm-thin Si_3_N_4_ membranes (Silson, UK), transparent to hard X-rays.

The Ethics committee at Lund University (Lund, Sweden) approved the study (permission number LU 275–90) and all participants gave their consent to the surgical procedure and subsequent morphological analysis after being provided with written and verbal information.

### 2.2. Experimental setup

**Figure 1:**
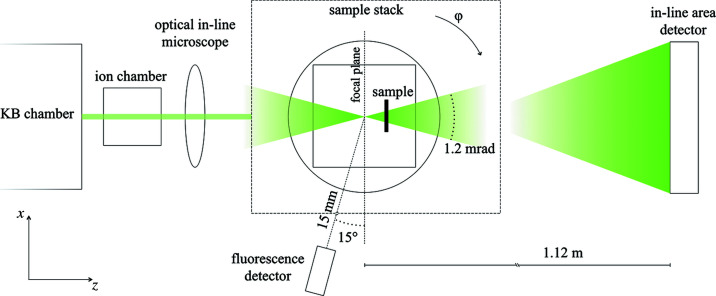
Schematic representation of the multimodal X-ray microscopy experimental setup. KB mirrors focus X-rays down to a focal spot of about 70 nm and with a 1.2 mrad divergence. The sample is mounted on a motor stack enabling *xyz* translation and θ, φ rotation, *i.e.* around the *x* and *y* axes, respectively. The sample can be translated in and out of the X-ray beam focus via translation along *z* (total travel range 46 mm), effectively tuning the X-ray beam size. An ion chamber is positioned downstream of the KB chamber to monitor the incoming X-ray photon flux. An optical in-line microscope provides a view onto the sample along the optical *z* axis. A fluorescence detector is positioned 15 mm away from focus, with a 15° orientation with respect to the focal plane. An in-line area detector is positioned 1.12 m downstream of the focus to collect holograms.

The experiment was carried out at the hard X-ray nano­focusing beamline NanoMAX at MAX IV (Johansson *et al.*, 2021 ▸). During the experiment, the photon energy was varied around 13 keV, as described in Section 2.3. A diagram of the experimental setup is represented in Fig. 1 ▸. A pair of Kirkpatrick–Baez (KB) mirrors focused the X-rays to *ca* 70 nm (68.5 nm at 13.0 keV). At the focal position, a two-circle goniometer was used for sample alignment and orientation, along with a stack of both coarse (20 mm stroke, 10 nm resolution, 100 nm repeatability) and fine (100 µm stroke, 1 nm resolution) *xyz* translation stages. Both step and continuous motion of the fine stages could be used to scan the sample in the transversal plane of the beam to generate 2D maps. For the collection of fluorescence spectra, a one-element silicon drift detector (SDD) (SiriusSD, RaySpec, UK) was used in combination with a high-performance pulse processor (Xspress3, Quantum Detectors, UK). The detector was positioned slightly upstream with respect to the focal plane, at a distance of 15 mm with a 15° orientation with respect to the transversal imaging plane.

In order to collect holography data, an additional translation stage and an area detector were added to the standard nXRF setup. The additional translation stage was a linear piezo stage (SLC-1740, SmarAct, Germany) with 26 mm stroke and 1 nm resolution. It was mounted on top of the motor stack and aligned with respect to the optical axis using the bottom goniometer. Together with the standard translation stages, this ensured a total travel range of 46 mm along the optical axis. The added area detector was a scientific complementary metal oxide semiconductor (sCMOS) camera system (Andor Zyla 4.2+, Oxford Instruments, UK) and was positioned 1.12 m downstream of the focal plane, along the beam path. The camera featured 2048 × 2048 pixels with an isotropic pixel size of 6.5 µm. It was combined with a 10 µm-thick LuAG:Ce scintillator and a 10× optical focusing lens system, leading to an effective pixel size dependent on the propagation distance of the divergent beam, *i.e.* the distance between the sample interaction plane and the scintillator, as discussed later.

Finally, an ion chamber was used to monitor the incoming X-ray photon flux and an adjustable optical in-line microscope was available for coarse sample navigation (resolution 3.3 µm at maximum magnification). The whole setup was in air, implying minor signal loss, especially for the softer X-ray fluorescence emission lines.

### 2.3. Data acquisition

For the multimodal microscopy scans, the energy of the photon beam was selected based on the Os *L*_1_ absorption edge, which is located at 13.0 keV. The beamline optics were optimized to maximize photon flux and minimize the focal spot size at 13.0 keV following NanoMAX’s standard ptychographic procedure (Björling *et al.*, 2020 ▸) and using the NanoMAX ‘high flux’ settings. By imaging a standard test pattern, an estimate of the focal spot size was produced which was in good agreement with the predicted value of 68.5 nm at 13.0 keV. In line with beamline specifications, the photon flux was energy-dependent.

In order to maximize contrast due to the relatively high electron density of Os, we decided to collect the holography data at a photon energy of 13.2 keV, above the Os *L*_1_ absorption edge. Conversely, we collected the fluorescence data below the same edge, at 12.8 keV, to take advantage of a slight increase in photon flux while sacrificing a few fluorescence emission lines of the abundant Os. Aside from Os – which was introduced as a staining to provide contrast for electron microscopy – the XRF spectra included several other elements of interest. Furthermore, for the high-resolution XRF scans, we decided to use a 200 µm defocused beam. The highest achievable resolving power based on the focused beam would have been 70 nm, but using a slightly larger beam we privileged the possibility of reducing absorbed dose and imaging larger fields of view, owing to the higher effective imaging speed (imaged area per unit time).

Once the experimental geometry was optimized, a fluorescence scan was performed for calibration purposes. We recorded fluorescence emission lines from Fe, Cu, La and Pb of a standard sample with known area mass density of each element (RF-200-0205-C00-X, AXO Dresden, Germany) using the same scanning parameters as for later high-resolution XRF scans. This allowed us to reproducibly retrieve the area mass density for each element present in the later XRF spectra.

The multimodal acquisition on the nerve tissue sample started with the overview holography scans. In order to optimize the phase reconstructions, holograms were recorded at four increasing defocusing distances (Zabler *et al.*, 2005 ▸): these were 30.00 mm, 31.64 mm, 33.32 mm and 35.02 mm, corresponding to beam sizes in the range 36–44 µm. For each *xy* position and defocusing distance, five holograms were collected with a 1 s exposure time. Each scan also recorded flat (*i.e.* direct beam) and dark frames to correct raw detector frames. For each sample, first a high-contrast feature away from any ROI was scanned to determine the effective pixel size associated to each distance of the defocusing series. Then, holography scans were performed on potential ROIs, based on the same defocusing series. The *xy* step size was adjusted to ensure at least some overlap between adjacent holograms for later alignment.

Once the overview images were obtained from the holography scans, ROIs could be identified on which to carry out high-resolution XRF scans. In order to produce fluorescence images with 200 nm spatial resolution, a scanning step size of 200 nm was used in combination with a 200 nm X-ray beam, achieved by shifting the sample back to a near-focus position, 200 µm downstream the focal plane. The scan was carried out in the *xy* plane, operating the motors in continuous motion for reduced overhead. At every scanning position a fluorescence spectrum was collected by the fluorescence detector with an exposure time of 100 ms.

## 3. Results

Based on the holograms collected on the high-contrast feature, the effective pixel size for each distance of the defocusing series was found to be 18.30 nm, 19.30 nm, 20.30 nm and 21.34 nm, respectively. This information was required to run the holographic reconstruction algorithms for each frame. First, contrast transfer function (CTF) retrieval (Guigay, 1977 ▸) was carried out. Then, the CTF results were used as the input of hybrid input–output (HIO) and error reduction (ER) (Gerchberg & Saxton, 1972 ▸; Fienup, 1982 ▸) iterative approaches within the *PyPhase* package (Langer *et al.*, 2021 ▸). The resulting phase-shift images were converted into units of electron density (Cloetens *et al.*, 1999 ▸) by assuming a homogeneous composition through the 2 µm-thick sample. Such conversion was only carried out in order to translate the recovered electron density from units of Å^−2^ to the more familiar Å^−3^ and it should not be taken to imply that the sample actually is homogeneous throughout its thickness. Frames within the same scans were stitched together based on their encoder-recorded motor positions, refined by image alignment among neighbouring frames.

**Figure 2:**
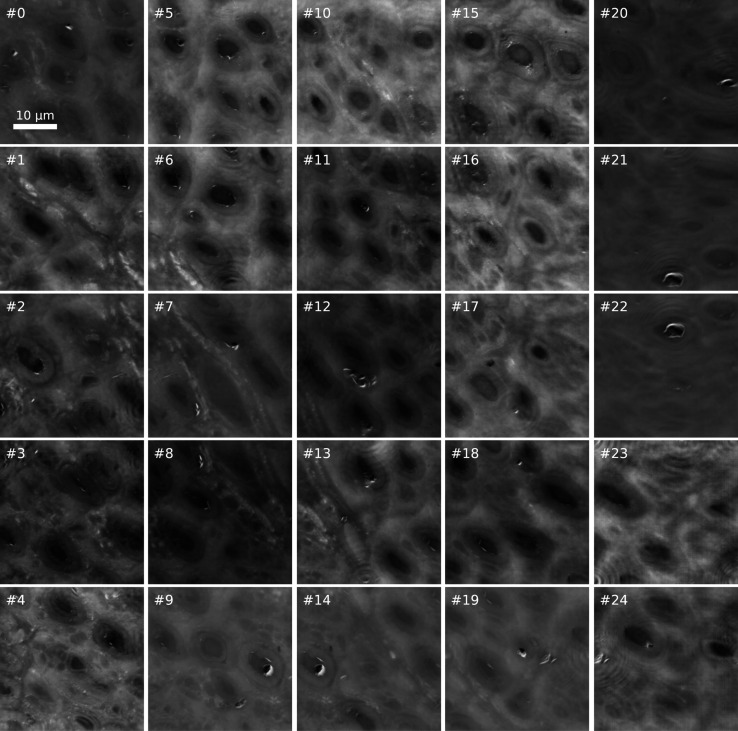
Preliminary reconstructions from a 5 × 5 holography scan covering a potential ROI of a section of an Os-stained human peripheral sural nerve biopsy from a healthy male. These qualitative results were used to delimit a ROI on which to perform a nanoscale X-ray fluorescence scan, as more conveniently annotated in Fig. 3 ▸. Further processing followed. The frame number is annotated on each frame, revealing the scanning sequence.

**Figure 3:**
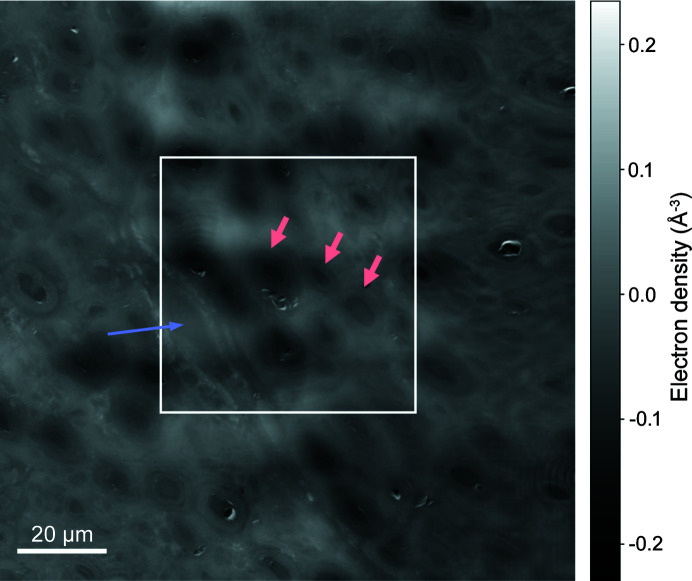
Quantitative result obtained from processing the images from Fig. 2 ▸. The ROI on which a nanoscale X-ray fluorescence scan was performed is highlighted by the white rectangle. A blood vessel (blue arrow) as well as myelin layers surrounding several axons (red arrows) can be identified. The intensity scale represents relative electron density variations and for convenience was converted into units of Å^−3^.

As an example of such holography acquisition pipeline, Fig. 2 ▸ shows the preliminary results of CTF retrieval run on each frame of a 5 × 5 scan performed as a 137 µm × 137 µm overview of a potential ROI. These qualitative unstitched images reveal a blood vessel as well as several Os-stained myelinated nerve fibres with a regular and dense distribution. The image quality was sufficient to delimit a ROI on which to perform a nXRF scan. Further processing led to the stitched relative electron density map shown in Fig. 3 ▸ where the white rectangle marks the nXRF ROI, selected to include at least part of the blood vessel and as many nerve fibres as possible. Exploiting the overlapping region of two adjacent frames, the achieved resolution was estimated to be 200 nm, based on Fourier ring correlation using the 1-bit threshold criterion (Van Heel & Schatz, 2005 ▸). This exceeded the 70 nm spatial resolution limit for this experiment, possibly because of minor stitching artefacts.

**Figure 4:**
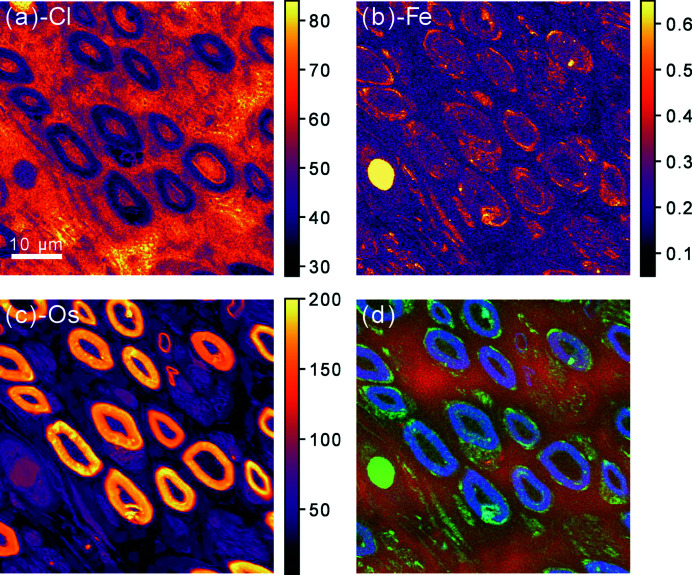
(*a*)–(*c*) Area mass density maps obtained via nanoscale X-ray fluorescence emission spectroscopy for (*a*) Cl, (*b*) Fe and (*c*) Os within a ROI of a section of an Os-stained human peripheral sural nerve biopsy from a healthy male. Linear intensity scales are shown in units of ng mm^−2^. (*d*) RGB representation of the same region including electron density information from the holography scan: red, green and blue channels represent electron density, Fe and Os area mass densities, respectively. A 10 µm scale bar for all images is given in (*a*).

XRF data were analysed using the Python-based software *PyMCA* (Solé *et al.*, 2007 ▸). A list of chemical elements expected to give rise to detectable fluorescence emission was produced taking into account the known chemical composition of the sample as well as the peaks observed within the raw fluorescence spectra. For each fluorescence scan, this list – along with other experimental parameters such as detector specifications, sample geometry, photon beam energy and scanning parameters – was fed into the *PyMCA* batch fitting algorithm. The algorithm is based on non-linear least-squares fitting of fluorescence emission lines and includes background subtraction. It returned quantitative fluorescence maps for each fitted line. Based on the calibration scan run on the standard sample, these maps were converted into absolute projected area mass density maps whose pixels represent values expressed in units of ng mm^−2^. This way, area mass density maps were obtained for Si, P, S, Cl, Ar, K, Ca, Ti, Fe, Ni, Zn and Os, of which Si was in the substrate (Si_3_N_4_), Ar in the air along the sample-to-detector path and Ni a known detector contamination. Based on the beam size and continuous-scan step size used, the resolution was estimated at 200 nm. Fig. 4 ▸ shows an example of area mass density maps obtained from a nXRF scan performed on the 60 µm × 60 µm ROI highlighted in Fig. 3 ▸. In this case the elements of interest were Cl, Fe and Os. As expected, Os was present throughout the sample, but its area mass density was particularly high in the myelin surrounding the axons, as intended by the initial staining process for morphometry (Sundkvist *et al.*, 2000 ▸; Mohseni *et al.*, 2017 ▸). The area mass density map of Fe clearly confirms the presence of a blood vessel and an Fe-rich blood cell therein. In the RGB representation, the electron density map from the holography scan is combined with area mass density maps from the fluorescence scan: red, green and blue channels are assigned to electron density, Fe and Os, respectively, in order to highlight co-localization. Principal component analysis (PCA) over the whole dataset for this ROI revealed that a single component accounted for 98% of the variance, with a dominant positive correlation between Os and P. On the other hand, only a weak negative correlation was found between Os, P and electron density, implying the latter was dependent on elements whose fluorescence emission was not measurable within this experimental setup, namely H, C, N and O, which together account for around 99 wt% in soft tissue.

## 4. Discussion

The localization of ROIs during synchrotron-based hard X-ray and high-resolution XRF experiments is typically achieved through low-resolution XRF scans within which a defocused beam is coarsely scanned across relatively wide areas of sample. For such overview scans, a balance is sought between an exposure time short enough to minimize potential radiation damage but long enough to provide sufficient contrast in the recovered coarse fluorescence map. Visible-light microscopy is sometimes used as an alternative approach. In that case, an in-line optical microscope is integrated with the beamline setup – as was the case for this experiment – and used for coarse sample navigation. This has the advantage of not inducing any radiation damage to the sample, but penetration depth is low and the achievable spatial resolution is typically limited to a few micrometres: 3.3 µm in this experiment. Furthermore, contrast can prove insufficient to resolve features of interest, as in the case of unstained biological samples. Using in-line holography instead offers the possibility to obtain overview images faster and with a lower dose than with XRF, while at the same time achieving spatial resolutions below 1 µm. It also entails the added value of a different X-ray imaging mode: in fact XRF scans generate elemental density maps of a few and distinct chemical elements, whereas in-line holography scans generate electron density maps which depend on the combination of all chemical elements, including those not revealed by XRF. These sets of different maps can be analysed to highlight correlations between morphology (*e.g.* structure) and chemistry (*e.g.* key elements) within samples. Hard X-ray experiments are often not designed or suited to measure fluorescence emission from low-*Z* elements (*Z* ≲ 14). With hard X-rays it then becomes particularly convenient to obtain electron density maps as these are the only means to infer the combined density distribution of low-*Z* elements (*e.g.* H, C, N, O) which account for the greatest mass fraction within organisms and many biogenic materials.

Our results fit within a wider clinical study in which these human nerve tissues were (Mohseni *et al.*, 2017 ▸) and are involved, and more measurements will be necessary to draw wider conclusions. However, and more importantly, these results demonstrate the viability of this powerful approach for any similar sample. In fact, nerve tissue is rather representative of most biological soft tissues with respect to X-rays. They all feature similar density (*ca* 1 g cm^−3^) and chemical composition (H, C, N and O account for *ca* 99 wt%) and their interaction with X-rays is thus similar, with more differences expected within the softer X-ray regime (*e.g.* below 2 keV). This also implies that the total absorbed dose within different soft tissues with similar thickness is similar. Given radiation sensitivity is expected to be comparable among soft tissues, this allows us to infer irradiation levels suited to prevent radiation damage at the relevant length scale. Within this experiment, no radiation damage was observed above the 200 nm image resolution after nXRF, meaning that any radiation-induced effect in the sample did not cause morphological alterations that could be resolved via holography. Therefore, we expect that other dehydrated soft tissues prepared in 2 µm-thin sections would also go through a similar 2D multimodal microscopy experiment undamaged.

The overview holography scan shown in Fig. 2 ▸ took just under 18 min, with an effective imaging speed of 17 µm^2^ s^−1^. The total dose absorbed by the sample throughout the scan was estimated at 110 kGy, based on the (1.65 ± 0.02) × 10^10^ photons s^−1^ incident photon flux measured by the ion chamber and the 13.2 keV incident photon energy. For comparison purposes, one could estimate the absorbed dose related to an overview scan carried out using fluorescence instead of holography. For example, the location of the ROI from Fig. 3 ▸ could have been determined via a coarse XRF scan exploiting a 1 µm defocused beam, thus producing an overview at a lower resolution than with holography, but still sufficient to resolve the stained axons. The experimental conditions would be the same as those of the XRF scan whose results are shown in Fig. 4 ▸, except that the coarse fluorescence scan would cover the same area as in Fig. 3 ▸ (137 µm × 137 µm) with a 1 µm step size. The total absorbed dose associated with such scan would be just over 1.0 MGy. Therefore, an overview fluorescence image covering the same area as the high-resolution holography scan, but with a resolution lower by a factor of five, would deliver a dose one order of magnitude higher. This highlights the twofold advantage of using holography for sample navigation.

Registration and optimization of the parameters of the holographic reconstruction algorithm used in this study required several hours in the early part of the experiment and were performed by experienced users. Though preliminary images of sufficient quality to identify ROIs suited for longer nXRF scans (*cf* Fig. 2 ▸) were available within minutes from the end of data acquisition, converged quantitative phase maps (*cf* Fig. 3 ▸) required a few hours of computation time. The continuous development of the *PyPhase* package (Langer *et al.*, 2021 ▸) will further increase user friendliness and enable novice users to generate converged holography reconstructions. However, it is expected that experiment design and setup will still require a solid understanding of holography and should hence be carried out by or in collaboration with experienced users or beamline staff.

On the other hand, the high-resolution nXRF scan used to obtain Fig. 4 ▸ took 168 min, with an effective imaging speed of 0.36 µm^2^ s^−1^. The absorbed dose was estimated at 30 MGy, based on the (2.47 ± 0.03) × 10^10^ photons s^−1^ incident photon flux measured by the ion chamber and the 12.8 keV incident photon energy. Similarly to holographic reconstructions, a few hours were needed before obtaining the quantitative area mass density maps from the XRF data. In this case, this was mainly due to the guided but manual process of labelling fluorescence emission lines and optimizing fitting parameters. The computation time for the final spectral fitting was on the order of a few minutes. However, qualitative elemental distribution maps were produced within minutes from the end of data acquisition and were used to assess the success of each scan and draw preliminary conclusions on different ROIs. Furthermore, since the time when the experiment was performed, further developments of the beamline control system were carried out which enabled live mapping based on pre-selected fluorescence emission lines (Björling *et al.*, 2021 ▸). The visualization of qualitative fluorescence maps while acquisition is ongoing contributes to further optimize the use of beam time.

Within the proposed experimental setup, fluorescence scans were carried out with the samples in near-focus position, thus exploiting beam sizes of up to a few hundred nanometres. Instead, for holography scans, samples were positioned well out of focus in the divergent beam (a few tens of millimetres) thus exploiting relatively large beam sizes (tens of micrometres). On the one hand, this caused holography scans to image areas two orders of magnitude faster than fluorescence scans, delivering images of similar resolution, within a comparable time scale after collection and via a different and complementary contrast mechanism. On the other hand, this also caused the holography scans to deliver two orders of magnitude less absorbed dose, thus significantly reducing the risk for radiation damage before high-resolution XRF. The dose delivered during such scans is usually constrained, primarily by the size of the features one aims at resolving and the concentration of the elements of interest therein. It is the combination of these factors that weighs most on the determination of the minimum amount of photons per unit area required to generate a detectable fluorescence signal. However, the proposed multimodal microscopy approach extends the tunability of the dose delivered during sample navigation and ROI selection. In such an experimental setup, even more pronounced trade-offs can be achieved between the two distinct imaging modes and the sample geometry can be flexibly adapted for both. This enables users to tune and optimize key experimental parameters such as field of view, image resolution and absorbed dose. For example, performing a high-resolution XRF scan within the same experimental setup but with the sample at the focal position would generate XRF images with 70 nm spatial resolution. Should the exposure time be kept unchanged, this would also cause a decrease in imaging speed and an increase in absorbed dose by one order of magnitude each, respectively. Conversely, performing a holography scan at a larger defocusing distance and hence beam size would increase the field of view of each hologram and hence increase imaging speed, though precisely by which factor heavily depends on the selected scanning routine (*e.g.* number of propagation distances, number of holograms per distance, overlap among adjacent holograms *etc*.). For most X-ray microscopy experiments, it is crucial to fine-tune absorbed dose, image resolution and field of view, and significant effort is often invested in finding a balance between them at the stage of experiment design and adapting it throughout the beam time. A limit for the maximum tolerable absorbed dose might be applied based on estimated or visible radiation damage. Similarly, the target spatial resolution could be set at, or just below, the expected size of the smallest feature of interest. Finally, the field of view might be chosen based on considerations on statistical representativeness as well as time availability.

The NanoMAX beamline has been designed as a nanofocus beamline and optimized to provide access to the nanoscale [<100 nm (Johansson *et al.*, 2021 ▸)]. This is a great benefit for XRF experiments whose ultimate resolution is limited by the beam size used on the sample. The experimental setup implemented within this work expands the range of accessible length scales further towards the microscale (>1 µm). Both in terms of spatial resolution and field of view, the combination of nXRF with in-line holography successfully bridges the gap between the nano- and microscale, *i.e.* the mesoscale (100 nm to 1 µm). Furthermore, it does so in a dose-efficient fashion and with an imaging speed higher than that of nXRF.

The addition of a rotation stage to the experimental setup presented enables collection of in-line holography projections suited for tomographic reconstruction, as recently demonstrated (Kalbfleisch *et al.*, 2022 ▸). Further setup optimization will reduce motor overhead and, in combination with the reduction of exposures at each defocusing distance, will allow us to increase the imaging speed by one order of magnitude, thus achieving 10^2^ µm^2^ s^−1^ in 2D and 10^2^ µm^3^ s^−1^ in 3D. Also, further development of the processing algorithms is expected to improve the spatial resolution achieved by holography and approach the limit of the focal spot size, which for the present experiment was 70 nm.

## 5. Conclusions

We demonstrated the combination of propagation-based in-line holography and X-ray fluorescence (XRF) emission spectroscopy as two complementary imaging modalities at a fourth-generation hard X-ray nanofocusing beamline. Both techniques were used within the same experiment, which was aimed at the 2D nanoscale characterization of sections from a human peripheral sural nerve biopsy. The samples were also representative of other biological soft tissue, thus demonstrating the wider applicability of the method. Holography was used to quickly and reliably identify ROIs within each sample. It produced electron density maps delivering morphological information with 200 nm resolution. XRF was used to produce quantitative area mass density maps for key elements with 200 nm resolution. XRF scans required relatively long measuring times and high absorbed doses compared with holography scans. Near-real-time preliminary results from the latter were exploited to optimize experimental parameters for the former, thus optimizing the use of limited beam time and minimizing absorbed doses for the samples.

This multimodal microscopy approach is expected to largely benefit users at NanoMAX as well as the wider synchrotron-based microscopy community. The performance of the experimental setup presented here is particularly relevant for biological samples, as for these the issue of identifying and optimizing ROIs while minimizing radiation damage is of great concern. This approach could also benefit samples from other fields of research, such as materials science and physical chemistry (Meirer & Weckhuysen, 2018 ▸), in which holography and XRF are already in use (Li *et al.*, 2019 ▸; Altenbach *et al.*, 2020 ▸; Veselý *et al.*, 2021 ▸).

This experiment was also the first successful demonstration of multimodal imaging combining in-line holography and fluorescence at NanoMAX, thus contributing to establish and expand the range of imaging techniques available at the beamline. We expect to extend this multimodal approach to 3D applications by combining the experimental setup presented with a high-precision rotation stage, suited for tomography experiments (Kalbfleisch *et al.*, 2022 ▸). Such experiments require longer measuring times and entail higher absorbed doses, so the flexible optimization of experimental parameters is crucial for their success, possibly along with the implementation of cryogenic sample cooling (Deng *et al.*, 2018 ▸; Conesa *et al.*, 2020 ▸; Gramaccioni *et al.*, 2020 ▸). Should radiation damage prevent the reliable collection of high-dose XRF tomography data, lower dose phase-contrast tomography could still be used to produce or validate quantitative results from 2D XRF scans on samples of variable thickness (Bardelli *et al.*, 2021 ▸).

Finally, our multimodal microscopy approach relies on the combination of a divergent nanofocused X-ray beam with translation of the sample along the optical axis. This is achieved with the addition of a longer translation stage which constitutes but a minor alteration to a standard hard X-ray nXRF imaging setup and yet greatly expands its range of accessible length scales, both in terms of imaging speed or field of view and achievable spatial resolution. We envision its implementation could be carried out at other similar microscopy beamlines making it easier for the user community to exploit their existing or potential multimodal imaging capabilities.
